# Supplementary material for: Radiation-Induced Overexpression of TGFβ and PODXL Contributes to Colorectal Cancer Cell Radioresistance through Enhanced Motility
Source: Cells. 2021 Aug 13;10(8):2087. doi: 10.3390/cells10082087 (PMC8393946; doi:10.3390/cells10082087)
Supplement: Supplementary file 1 [file cells-10-02087-s001.zip › cells-1282159-SI.pdf]

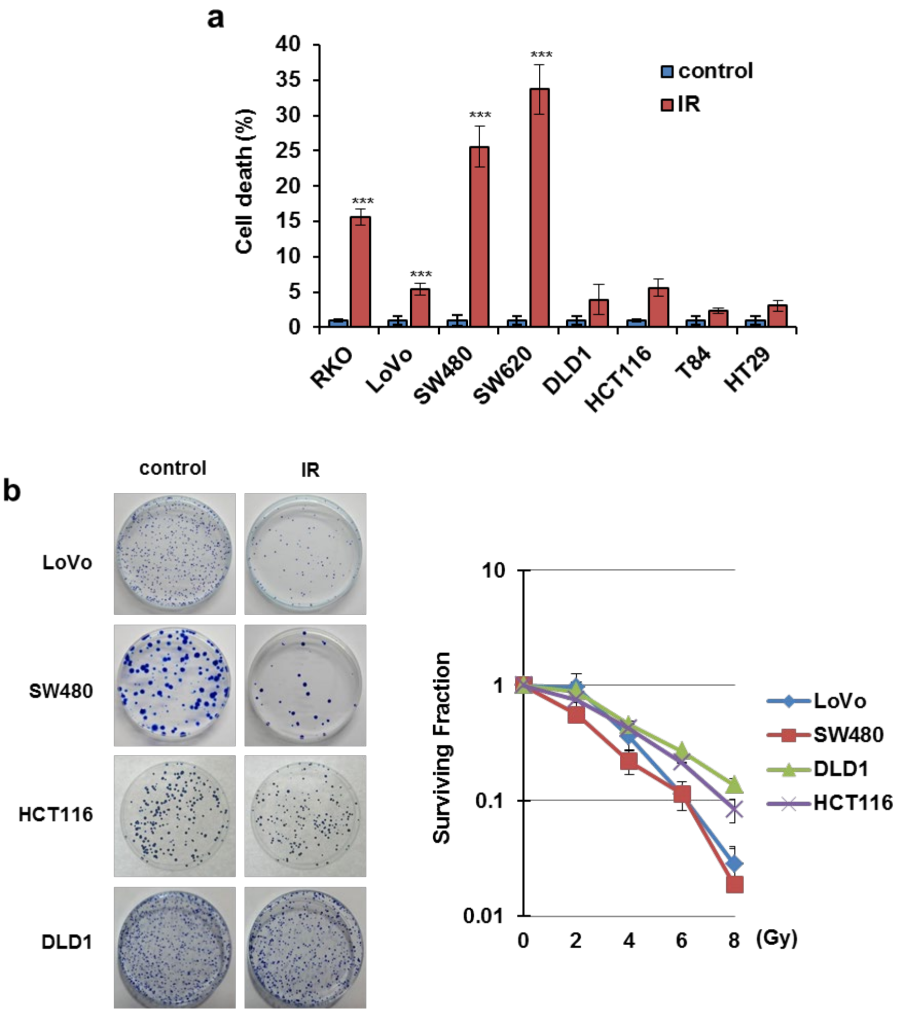

Figure S1. IR exposure affects CRC cell growth and viability. (a) Eight colorectal cancer cell lines were exposed to IR at 5 Gy. Twenty-four hours after IR exposure, cell viability was measured by CCK-8 assay (b) LoVo, SW480, HCT116 and DLD1 were exposed to the indicated doses of IR and then incubated for two weeks. Colony number was measured from three replicate plates of three independent experiments (bars indicate SD). Representative images from two weeks after plating are shown.

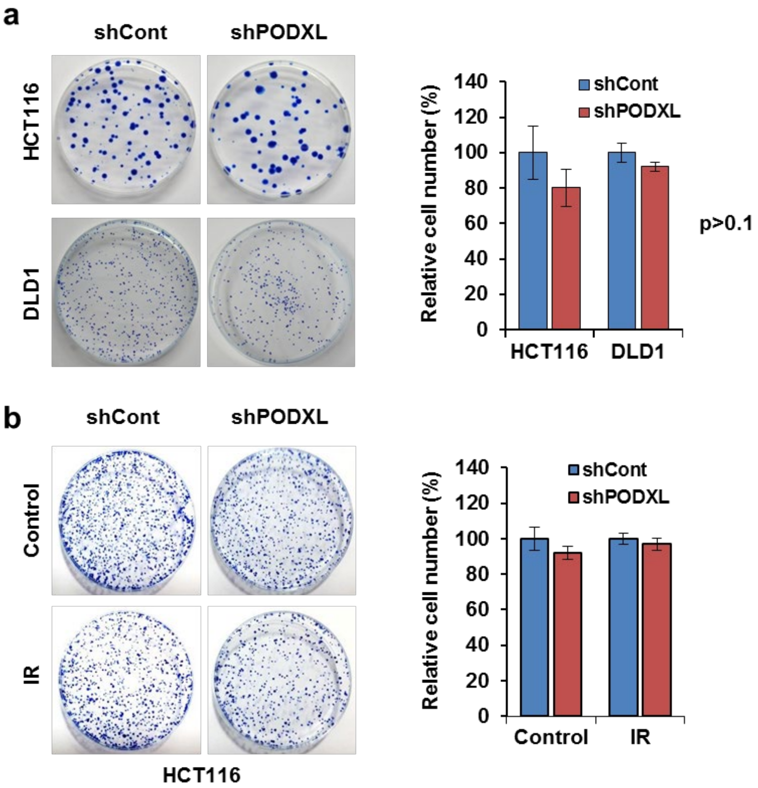

Figure S2. PODXL could not suppress cell growth of CRC cells. (a) shCont- or shPODXL transfected HCT116 and DLD1 cells were maintained for 21 days. (b)shCont and shPODXL cells were exposed to IR (5 Gy) and maintained for 21 days. The Colony number was calculated from three replicate plates of three independent experiments; bars indicate SD. Representative images from 21 days post-plating are shown.

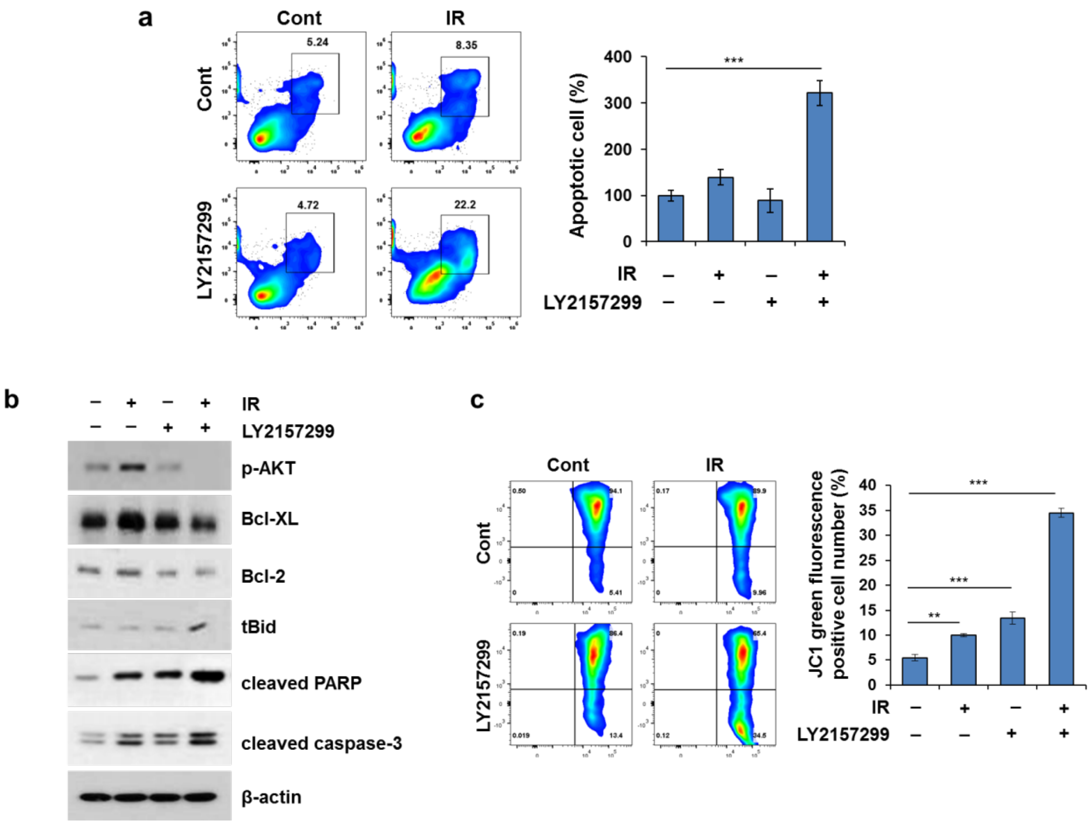

Figure S3. Combined galunisertib and IR treatment induced apoptosis in CRC cells. HCT116 cells were treated with galunisertib (10 μmol/l) alone, IR (5 Gy) alone, or combined for 24 h (a) Representative images of flow-cytometry analysis of apoptosis. The graph represents relative apoptotic cells. ***p < 0.005. (b) Western-blot analysis was used to measured p-AKT, Bcl-2, Bcl-xL, tBid, cleaved caspase-3 and cleaved PARP expressions. (c) Representative images of flow-cytometry analysis of mitochondrial potential. HCT116 cells were stained with JC-1 for 30 min and measured by flow cytometry. Data were statistically compared (right). Data were represented as the mean ± SD obtained from three independent experiments. *p < 0.05; **p < 0.01. The graph represents loss of mitochondrial potential (%). ***p < 0.005..
